# Challenges in delivery of skilled maternal care – experiences of community midwives in Pakistan

**DOI:** 10.1186/1471-2393-14-59

**Published:** 2014-02-05

**Authors:** Mariyam Sarfraz, Saima Hamid

**Affiliations:** 1 Health Services Academy, Chak Shehzad, Islamabad 44000, Pakistan

## Abstract

**Background:**

Maternal mortality ratio in Pakistan remains high at 276 per 100000 live births (175 in the urban areas and 319 in rural) with a mother dying as a result of giving birth every 20 minutes. Despite the intervening years since the Safe Motherhood Initiative launch and the Millennium Development Goals (MDGs), there have been few improvements in MDGs 4 and 5 in Pakistan. A key underlying reason is that only 39% of the births are attended by skilled birth attendants. Pakistan, like many other developing countries has been struggling to make improvements in maternal and neonatal health, amongst other measures, which include a nationwide health infrastructure network. Recently, government of Pakistan revised its maternal and newborn health program and introduced a new cadre of community based birth attendants, called community midwives (CMW), trained to conduct home-based deliveries. There is limited research available on field experiences of community midwives as maternal health care providers. Formative research was designed and conducted in a rural district of Pakistan with the objective of exploring role of CMWs as home based skilled service providers and the challenges they face in provision of skilled maternal care.

**Methods:**

A qualitative research using content analysis was conducted in one rural district (Attock) of Pakistan. Focus group discussions were conducted with CMWs and other community based health workers as LHWs and LHSs, focusing on the role of CMWs in the existing primary health care infrastructure.

**Results:**

Results of this study reveal that the community midwives are struggling for survival in rural areas as maternal care providers as they are inadequately trained, lack sufficient resources to deliver services in their catchment areas and lack facilitation for integration in district health system.

**Conclusions:**

CMWs face many challenges in the field related to the communities' attitude and the health system. With adequate training and facilitation by health department, CMWs have potential to play a vital role in reducing burden of maternal morbidity and in achieving significant gains in improving maternal and child health.

## Background

Maternal mortality is one of the greatest health and development challenges facing the world, especially in the developing world where maternal mortality ratios have barely fallen in the last 50 years, even as other health indicators have improved [1]. The maternal mortality ratio in Pakistan remains high at 276 per 100000 live births (175 in urban areas and 319 in rural) [2] with a mother dying as a result of giving birth every 20 minutes. Despite the intervening years since Safe Motherhood Initiative launch in 1987 and the Millennium Development Goals (MDGs), there have been few improvements in MDGs 4 and 5 in Pakistan [3]. The maternal mortality rate in Pakistan was estimated to be 533 in 1993, 276 in 2007 [4] and 260 in 2008 [5]. The current rate of 276 shows a reduction of 49% in a span of 20 years, however, it is estimated that at the current rate of progress, Pakistan will not achieve the MDG target of 140 by 2015 [6]. An examination of the Pakistan Demographic and Health Survey (PDHS) 1991 and 2006 data reveals that more than 50% of women prefer a traditional birth attendant’s (TBA’s) assistance for delivery rather than a skilled care provider [4,7]. Of all the births, 39% are attended by skilled birth attendants and 34% take place in a health facility [8].

Community based programs for improving births attended by skilled attendants and reducing maternal and new born mortality have been introduced in several developing countries such as Sri Lanka, India, Indonesia and African countries. Success of such initiatives depends on several factors such as a well-developed health system, strong referral systems and linkages, availability of transport networks and emergency services. The midwifery programs there have met with varying levels of success [9-12] in reducing maternal mortality through increased skilled birth attendance. The State of World’s Midwifery report has identified that most of the developing countries do not have enough qualified midwives and birth attendants for managing the high number of pregnancies, 15 percent of which result in obstetric complications [13]. Several countries, including Pakistan, need to increase their midwifery work force multiple times while also improving quality of services, providing a wider coverage, streamlining referral system and strengthening the overall existing health system.

Pakistan has been struggling to make improvements in maternal and neonatal health amongst other measures. This includes a nationwide health infrastructure network with thousands of first level care facilities and community based health workers for providing maternal and child health care to the rural populations. A cadre of Lady Health Visitors (LHV), introduced in 1955, were the first community based midwives [14]. The ministry of health also trained several traditional birth attendants for providing and improving maternal and child care. In 1994, another cadre of community based health workers, the Lady Health Workers (LHWs) were created to provide health education, antenatal care, immunization services, referral linkages, family planning services and basic curative care. The program has been successful in providing basic health education and care to rural communities through 100,000 health workers but the intended impact in increasing skilled attendance at deliveries [15] in rural areas of Pakistan has not been achieved. More recently, government of Pakistan revised its maternal and new born health program and introduced a new cadre of community based skilled birth attendants, called community midwives (CMWs), trained to conduct home-based deliveries in 2006 [16]. Details of the community based health workers’ programs and trainings are given in Table 1. To increase skilled birth attendance in rural areas and underserved urban slums, the Program besides focusing on health system strengthening for providing emergency obstetric care services, aimed to deploy 12,000 community midwives (CMWs) in community of their residence by the year 2012.

**Table 1:** Characteristics of health workers’ training program

**Health worker**	**Program and Year of initiation**	**Funded by**	**Selection Criteria**	**Training duration**
LHW [17]	National Program for Family Planning and Primary Health Care (NPFP&PHC); 1994	Government of Pakistan	Aged 18 to 45 years; Preferably married; At least 8 years of schooling; Resident of catchment area	15 months (facility and community based training)
LHS [18]			Aged 22 – 45 years; Preferably married; At least 12 years of education; Resident of catchment area	12 Months (facility and community based training)
CMW [19]	MNCH Program; 2006	Government of Pakistan and UNFPA	Aged 18 – 35; Preferably married; At least 10 years of education (science subjects); Resident of catchment area	18 Months (training at nursing council accredited midwifery schools)

Despite a large national and donor investment, national level household surveys [2,20] and research evidence from several rural regions of Pakistan [21-23] suggests that the utilization of maternal care through community midwives is still very low and the maternal health indicators have not shown significant improvements so far. Both these programs of LHWs and CMWs were vertical health care programs till 2011, under control of federal ministry of health and not integrated with the provincial health systems. The health systems in Pakistan recently underwent a reform of devolution of Federal Health Ministry which has effectively abolished the central ministry functions and transferred them to the provinces. However, the fate of the vertical programs is not defined and they will be managed by the Planning Commission till 2014 [24,25].

There is inadequate evidence available on the experiences of the community midwives as maternal health care providers. A qualitative study was conducted in one rural district (Attock) of Pakistan to explain the role of the CMWs and the challenges faced by them in the provision of skilled care by exploring perspectives of CMWs and other community based workers as LHWs and LHSs. This paper seeks to explain the programmatic and cultural barriers and constraints faced by CMWs in delivery of maternal care to rural communities.

## Methods

### Study site

The study was conducted in rural tehsils^a^ of District Attock (northern Punjab) in May 2011. District Attock has six tehsils, 5 rural and one urban; one of the rural tehsils was excluded as it did not have any CMWs deployed at time of the study. The terrain in rural Attock is mountainous, has an underdeveloped road network and public transport system. The selected site has geographical and social characteristics similar to most of northern Punjab and some areas of Khyber Pakhtunkhwa. The total population of the district is 1.58 million scattered over vast areas; the rural to urban distribution is 80 percent vs. 20 percent. The district has 6 large urban areas, 75 union councils and 440 villages. Literacy rate is 49.3 percent with 67 percent males and 32 percent female literacy. Farming and livestock rearing are the main economic activities in the district [26]. The MNCH program in District Attock is funded and managed by the Government of Pakistan with no additional donor support and hence has resource constraints reflective of situation in most of the districts of Pakistan. This unique situation of the MNCH program made this area an ideal location for exploring the challenges faced by community based health workers.

### Health profile

The public-sector healthcare infrastructure in the district includes: 1 District Headquarter (DHQ) hospital, 5 Tehsil Headquarter (THQ) hospitals, and 5 Rural Health Centers (RHC), 57 Basic Health Units (BHU), 7 Maternal and Child Health (MCH) centers and 3 sub-health centers. The district has 951 Lady Health Workers (LHWs) and 41 Lady Health Supervisors (LHSs), covering 57 percent of the population [26]. At the time of this study, 33 trained CMWs had been deployed in the district since 2009, for provision of midwifery services [27]. A Public Health Nursing School affiliated with the DHQ trains nurses, Lady Health Visitors (LHVs) and Community Midwives (CMWs). The clinical training of the CMWs is conducted at secondary or tertiary level facilities by obstetricians, with other trainees including Nurses and post graduate trainee doctors. There is also a vast network of private healthcare providers in Attock including doctors, pharmacists, traditional and informal, untrained medicine practitioners.

The maternal mortality rate for Punjab is 278 per 100,000 live births and IMR is 45 per thousand live births. Antenatal care is utilized by 55.7% of the rural population and skilled attendance at delivery is 43% [26]. Antenatal care is provided by a variety of local providers which include medical doctor (50%), LHV (5%), LHW (0.8%) and Traditional Birth Attendant (TBA) (32%) [26]. Deliveries are conducted mainly by doctors (40%) and TBAs (50.6%) [28].

### Study design and data collection

The research team comprised of three medical doctors (PI, Co-PI and technical advisor) with specialization in maternal health, anthropology and public health. The study objectives required flexible qualitative tools for developing an understanding of experiences of community based health workers, particularly CMWs. The research team reviewed the deployment guidelines of community based health workers and developed field guides based on their decreed roles and responsibilities. The field plan for data collection was developed in consultation with the program officials based in District Attock. Focus group discussions were then conducted with homogenous groups of community based maternal health care providers^b^ in rural areas of District Attock to explore the experiences of the recently deployed CMWs. Three cadres of community based health workers (CMWs, LHWs and LHSs) were selected as study participants since all are working within the primary health care system. The field guides developed were semi-structured, with open-ended questions, covering domains such as the role and responsibilities of CMWs, their satisfaction with their training, experience in the field, challenges faced in service delivery and any suggestions for improving their work conditions. The discussions were conducted in the local Punjabi dialect of *Pothwari*. Discussions were audio taped with participants’ permission.

The participants were encouraged by Moderator (PI) to interact with each other and comment on others’ experiences as group process can help in exploring and clarifying their views [29]. This allowed the participants to respond in a way that reflected their perceptions and experiences regarding community based maternal health care provided by CMWs. The observer (Co-PI) noted non-verbal interactions of the group while a note keeper (data collector) kept a log of proceedings of discussions in case there were any technical failures in recordings.

Field notes were taken during and immediately after each session by the data collection team. Transcripts were later developed in English for further analysis. For quality control, information collected through note-taking was cross-checked for completeness and consistency before and during data processing by the research team.

### Study participants

All the deployed community based maternal health care providers in rural areas of District Attock were included i.e. CMWs, LHWs, and LHSs. The roles and responsibilities of the community health workers are given in Table 2.

**Table 2:** Roles and responsibilities of community health workers

**Health worker**	**Roles and Responsibilities**
LHW [17]	Act as liaison between formal health system and community; Disseminate health education messages; Provide contraceptives; Undertake nutritional interventions and emphasize on breast feeding; Coordinate with EPI (extended program of immunization) for immunization of mothers and children against vaccine preventable diseases
LHS [18]	Provide supervisory support to LHWs; Conduct on the job training; Ensure quality performance by the LHWs
CMW [19]	Provide individualized care to the pregnant women in her own environment and help her in self-care; Provide guidance and counseling to the community for healthy habits, and involve the family in preparation for childbirth and for unforeseen emergencies; Identify actual or anticipated conditions requiring medical attention and make timely referrals

Purposive sampling was used, inviting the participation of those community based providers who had been serving actively in their local communities for the last five years; these health workers were identified with help of district program coordinators of the MNCH and National Program. The number of participants in all FDGs is detailed in Table 3.

**Table 3:** Total number of FGDs and Participants

**Cadre**	**No. of FGDs**	**Total no. of participants**	**Area**
CMW	3	16	Mian Wala, Bahter, Pind Sultani (Jand)
LHW	4	27	Bahter, Kot Sunki (Hassan Abdal), Pind Sultani (Jand)
LHS	3	30	Public Health Nursing School, Attock

Each participant was given verbal information about the study by the research team and was given a consent form prior to participation. All participants were invited for one FDG only. The FGDs with CMWs and LHWs were organized and conducted in the community; prior to FGDs, participants were asked to identify a location convenient to them and discussion session was organized in home of health worker in that area. The FGDs with the LHS were conducted in premises of district program office where all 30 LHS had convened for a monthly meeting. No monetary compensation was given to any of the FGD participants; however, light refreshments were arranged for them. FGDs were preferred over individual interviews as the researchers were not only interested in individual experiences but felt that in a homogenous group, discussions would be rich and even sensitive issues discussed openly. Furthermore this method allowed research team to get input from a bigger number of participants in a short period of time. The participants were informed that participation was voluntary and they had the right to withdraw from the study at any time. In each of the FGD, topic guide was followed loosely, allowing group interaction to decide which topics were discussed first. If the participants did not talk about an important issue, key questions using probes were asked to encourage discussion. Each session fed into the next one, with key issues or outstanding questions taken forward into the subsequent FGDs. Ethical clearance for the study was obtained from the Pakistan Medical and Research Council.

### Data analysis

A qualitative content analysis was carried out for an inductive analysis of thematic content with category construction based on analysis of transcripts of participants’ opinions. This inductive approach is a systematic procedure to analyze qualitative data in which identified themes emerge from a group of categories with common meanings. Analysis of the transcripts was done manually. As a first step transcripts were read multiple times by the authors. The authors independently identified the meaning units, followed by coding and their categorization. The research team then jointly reviewed their work and through a consensus identified the underlying sub-themes and theme [30,31]. Trustworthiness of the results was ensured by involvement of research team throughout the analysis process. The results were also shared with the participants for validation. The authors also had the results peer reviewed by two independent researchers having an experience of qualitative research.

## Results

### Characteristics of participants

In all, 10 FGDs with CMWs, LHWs and LHSs were conducted. Ten participants were invited to each FGD and about 5 to 10 participated in each group discussion. Of the total 1025 community health workers in Attock District (LHW – 962, LHS – 30, CMW – 33), 73 participated in this study. The ages of the participants ranged from 18 years to 45 years. Majority of the CMWs were in their twenties, unmarried and were relatively younger than the other cadres of health workers (Table 4). The oldest CMW was 32 years of age and was married. All of the LHSs had a graduate degree and were married. The educational qualifications of the LHWs varied from grade 8 to grade 12. Amongst the LHWs, the younger LHWs were unmarried.

**Table 4:** Age groups of participants

**Health worker cadre**	**Age groups**	**Work experience (range in years)**
CMWs	19 - 32	2 years
LHWs	18 - 45	2 – 17 years
LHS	28 - 42	5 to 17 years

### Findings

The overarching theme emerging from our analysis is that ‘Community Midwives are Struggling for Survival’ as qualified home based maternal health care providers. From the time of induction they were not prepared for working in community settings and providing door to door services. This was evident by lack of provision of appropriate equipment for their use in field, minimal financial and logistics support from health department, and lack of facilitation from LHWs and untrained TBAs. Besides the inherent training problems, in the community CMWs had competitors, which included the well-established local, untrained traditional birth attendant (Dai), and Lady Health Visitors (LHV). The Lady Health Worker (LHW), a government deployed community based health worker, entrusted with the responsibility of introducing CMW and sharing information about pregnant women registered with them, did not extend her full facilitation to CMWs in their jurisdiction as they were already required by the health department to refer pregnant clients to LHVs for deliveries.

*“The program first introduced me to LHWs and they all said that they would help me but they never did. I visited the community by myself and people told me that the LHW asked them not to cooperate with you. The salary I get from program is only Rs. 2000 which I use for medicines or transport. Village women go to LHV or dai for delivery. I want to leave the program as soon as the service bond of three year period is finished. But then I think that time heals all wounds and these problems will be over too. (Waqt sub say bara murhum hai. Waqt kay saath yeh masly saray hal hongay)”* (CMW)

The main theme is supported by two sub themes: Flawed planning and implementation of the Program, and Teething problems in field. Analysis process from categories to sub-themes and main theme is given in Table 5.

**Table 5:** Analysis results – themes and categories derived from the data

**Theme**	**Sub theme**	**Category**
***CMWs struggling for survival***	**Flawed planning and implementation of program**	Compromised induction and training process
		Incomplete and deficient program implementation
	**Program facing teething problems in field**	Harsh field conditions
		Uncooperative and perverse community attitude
		Harassment by community and health system
		Competition for service provision
		CMWs’ aspirations for facility based jobs

### Flawed planning and implementation of program

The first sub-theme explains the trainee midwives’ experiences during training at schools of midwifery. Where they were trained by nursing instructors in class rooms ill-equipped for training students in midwifery skills for home based care. Trainees were given shared hostel accommodations with nursing school students where the midwifery students were treated as subordinates. Also, the hostel accommodations had no facilities for married women and their children resulting in their dropout. Following training, deployment process for the midwives was neither defined nor were they provided with any guidelines.

The three categories explaining this sub-theme are described hereunder:

### Compromised induction and training process

The scope of work required of a community midwife was not highlighted in program admission advertisements and selected trainees were briefed about their role and responsibilities much later in training. The CMWs interviewed carried the impression that following their training they would be appointed at a local primary healthcare facility with access to support facilities for delivery of services in local community. This is reflected in the following quote by a CMW inducted in 2008.

*“The Nazim of our area told me that, “you will get a job in a Basic Health Unit (BHU), car for field visits and a pay of about 10,000 rupees”. So I joined the program but after I had joined the training, we were told that we had to work in the community.” (*CMW)

Following their induction into the program, the training of the CMWs did not conform to that proposed in the PC-1 due to restricted funding and lack of trained midwifery tutors. The CMWs spent most of their practical training time filling in for the deficient nursing staff at THQ or DHQ.

*“We were not allowed to attend to the patients in labor room. The nursing staff used to say that if you give us a treat then only we would let you work here. We had to spend 1000 to 1500 rupees for some snacks then only would they let us conduct the deliveries. The doctors there are very nice but the nursing staff gave us tough time.”* (CMW)

The trainee CMWs shared hostel accommodation with other nursing school students. The facilities not being exclusive for CMWs were inadequate, with confined living quarters, lacked amenities such as a cafeteria and laundry services; a serious flaw was that the program did not factor in the requirements of married students – there was no accommodation for children in the hostel, resulting in a mass departure of married trainees.

*“Many girls who joined the training were married but as they were not allowed to keep their children with them, they left”* (CMW)

#### Incomplete and deficient program implementation

Initially, CMWs were attached to their local BHU for one year and were required to perform duties as required by the doctor in-charge, without any stipend or salary. In 2010, CMWs were issued a letter by the MNCH Punjab Coordinator identifying their duties and entitlement to a stipend of PKR 2000 (~USD 25). They were also given a clean delivery kit, family planning supplies and were asked to designate a room in their homes as a clinic for conducting midwifery duties

When asked about the challenges faced in delivering duties assigned, CMWs highlighted several difficulties. Covering the catchment population of 10,000 required a young, unmarried CMW from a conservative rural background to travel across vast areas. The public transport available did not cover all areas within catchment population, requiring the CMW to use multiple modes of transport such as a van, car and motorbike, besides walking for miles.

*“We have to cover a population of 10,000. We live in one village and our access to other areas is very difficult because the villages are scattered. Some areas are at a distance of 5–6 Km and no public transport is available to go there, in that case we have to rent a private car or a taxi.”* (CMW)

Equipment and medicines given to the CMW were not sufficient for providing maternal health services to her catchment population which reflected poorly on her role as a community based health care provider. CMWs did not have resuscitation equipment for a new born baby, or an anesthetic or sutures for stitching tears/cuts. Patients requiring such services had to be referred to a health facility, which was an inconvenience for the villagers. CMW were also provided a limited range of family planning supplies (one time only) which mostly included condoms, few packets of oral contraceptive pills and one injectable contraceptive for all of the catchment population. CMWs had not been provided any education material either for health education, awareness raising and community mobilization.

*“People say that LHW gives them medicines but the CMW has nothing to give them so it doesn’t matter if she visits or not (Aaway, na aaway, ki farak painda; ou koi dawaiyan asan no daindee payee)”* (LHS)

The monthly stipend was mostly used by CMWs to cover travel expenses in their catchment population and purchasing various medicines required for delivery of their services. The monthly stipends were also frequently delayed.

*“Our pay is only 2000 rupees. No one in other program is getting that low pay. If we refer a case, the patients ask us to go with them and that cost us 1000 to 1500 rupees for transportation. How can we survive in 2000 rupees? 2000 rupees which we get are spent mostly on traveling and transport to Attock for monthly meetings.”* (CMW)

Field supervision of the CMWs was not integrated well within the health system. Technical supervision of CMWs was done by tutors from the training school and administrative supervision was responsibility LHS who was also supervising LHWs. LHS were not given an added incentives for additional workload and considered this task a burden.

*“I was only told that I have to visit the CMW in my area but they did not tell me why I should visit her, what should I check her for and how to do that. If they tell me their problems such as lack of equipment/materials or trouble with community, I cannot help them as I have not been given any specific instructions regarding them. We have to supervise LHWs and now we have to cover CMWs as well, but our pay is the same”* (LHS)

A grievance redressal system for the CMWs was not defined. The District MNCH officials catered to complaints/field problems within their purview and power but the feedback system on complaints filed was weak.

### CMW Program facing teething problems in field

In the FGDs, CMWs discussed the challenges they faced in delivering their services and identified problems for which the program was not supporting them. The CMWs categorized their problems as teething problems which they thought would eventually reduce with time as it did with the Lady Health Workers, who have worked in the community for several years. The CMWs also expressed an eagerness to take up a facility based job as soon as their service bond with the program ended because of low salary and deficient field support. The categories contributing to this sub-theme are detailed here under.

### Harsh field conditions

Following the completion of their midwifery training, the CMWs were required to sign a service bond of three years’ duration, whereby, they pledged an amount of approximately PKR 300000 for service in community and their diplomas were also held in escrow for the duration specified. This service bond allowed the MNCH program to exercise control on the deployed CMWs to continue working in the community for providing maternal health services. Almost all the CMWs belonged to poor, rural households, with barely enough savings in cash; this bond was a continuous strain on the CMWs and their families. The CMWs could not take up gainful employment elsewhere and were under pressure from their family to complete the service bond period.

“My family doesn’t allow me to go alone, one person always accompanies me. Sometimes I have to go twice or thrice in a day to visit the community and I have to bear costs for both our transportation. I want to stop working as CMW but my father does not allow me to do so as I still have one year left on the period of the bond. We cannot afford to pay the program back the amount pledged in the bond.” (CMW)

Most of the CMWs interviewed expressed that they had joined the program to earn and provide financial support for their families. However, due to the extensive scope of work, deficient support and insufficient stipend, they were instead dependent on financial support from their families for carrying out their duties. The CMWs hence considered themselves and their work an added burden on the family’s limited resources. Moreover, during their field visits they needed to be accompanied by a male member of the family or an elder female which incurred additional travel costs and also loss of daily wage of the accompanying escort. The young CMWs were under immense pressures from the family to complete their service bond period and quit their jobs.

*“I received a call for assisting in a delivery at 2.30 am in winter and went there but they didn’t pay me anything. They had nothing to pay so I didn’t ask again. It is difficult to leave my young daughter home and take my husband, this affects his work too and then we don’t get anything in return. We don’t have enough resources. I am not doing it as a hobby. I am needy, I thought that this would help me financially and I would also be able to help others”* (CMW)

Management issues, particularly those pertaining to field support were raised by all the respondents. The program had not provided the CMWs with any transport facility for covering her designated jurisdiction, neither was there a compensation mechanism for reimbursing them for the expenses incurred while working. Some clients had to be referred to a higher level facility, however, the public sector facility was either very far from the patient’s home or there was no doctor available there for providing services. This reflected poorly on the CMWs’ professionalism and her role was further undermined by the low quality of clinical services given to the referred patients.

*“I had to refer two babies to private doctor because I had nothing to resuscitate them with. No senior doctor is available in the THQ in the afternoon or in the night and Attock is very far from our area. When they (client family) came back they complained that we spent too much because of me. We should have all the necessary equipment in our clinic. This is not good for my reputation.”* (CMW)

### Uncooperative and perverse community attitude

Most of our respondents had experienced a hostile and unsociable response from the community in response to their work. The CMW, by virtue of her being young and unmarried, a *bachi (a young girl)*, was considered by community members ineligible for providing maternal care services. Respondents expressed that a common perception amongst rural communities was that as they are unmarried, they were as yet naïve to the reproductive health matters of married women. The CMWs felt frustrated that community women did not discuss their problems with them and respected the local TBA’s opinion more as she was considered to have more experience in these matters.

*“On our face, they acknowledge and commit that they will get delivered by us but then they call the TBA. When we ask them, they tell us that our mothers-in-law said that “She is a very young girl, what would she know. Dai has more experience in this matter, call her”* (CMW)

Some of the CMWs shared that for building trust of the community in their abilities, they take along with them a married, elder woman, such as their mother or married sister, who encourage women to talk to them about their problems. This trust building technique was found to be useful as the women then discussed their problems with the married relative who relayed it to CMW for further necessary advice or action. The married CMWs, on the other hand, did not face such problems as being married qualified them as being knowledgeable of information on women’s reproductive health issues.

*“I am married for 1 and ½ years now. When I was unmarried people used to say that* ‘*she is young, unmarried girl, what would she know and it will be harmful for our daughter-in-law or the baby’. After my marriage, now when I tell them something they understand it and also discuss their problems with me comfortably. Now they know that I know everything but when I was unmarried it was very hard to make them understand that we have done the training and know about maternal health. Now it is better”* (CMW)

Many of the rural community members did not allow CMWs to enter their homes for fear that they might cast an evil eye on pregnant women or new born child. Similarly, they also believed that the CMW could bring some evil spirits in their home, harming the new born child.

*“The people are so superstitious that they don’t let us see their babies or the pregnant women. They say “evil spirits get attached to our baby and you may cast an evil eye”. Some say that after we take measurements of their pregnant daughter, she starts losing weight as we do some magic/voodoo on her”* (CMW)

CMWs who did gain access to a prospective client’s home were faced with problem of their expectation for free services i.e. antenatal checkups, delivery and medicines will be free. This attitude has developed in rural communities in wake of LHWs’ practice who provide free medicines for common ailments to rural community members. Another reason for not giving any fee to CMW was that as she was a resident of the same *mohalla*^*c*^/village, she was expected to give free services to her own *biraadari walay*^*d*^ and asking for fee was frowned upon. In some women’s homes, the CMWs were told that since they had come uninvited and by their own choice, they should not expect the family to pay for the services they provide.

Local communities also considered CMWs same as untrained TBAs, which was a cause of embarrassment for most CMWs. 

*“People know that we are trained, but they still say that you’ve done TBA training (aap nay daioun wala course kia hua hay). It is indeed very embarrassing for us when somebody calls you a “dai”. Although madam, it should not be embarrassing, because if someone learns to do something, they do it for a purpose. Now those illiterate women (dais), with no formal training are also doing the same job, but people do not understand this”* (CMW)

This attitude of this community towards CMW had been corroborated by the LHWs and LHSs. They expressed that since community midwives were relatively new in the field rural communities would take time to trust the new-comers for health matters, especially a young girl for care of the pregnant women and newborn children.

### Harassment by community and health system

Abuse and harassment of the CMWs by community members and other community based health workers was evident in discussions with respondents. This behavior and attitude had demotivated the young CMWs, and compromised their ability to maintain professionalism in the community where they worked. A particularly serious dimension was harassment perpetrated by the community members and other health workers, both while in training and in field after deployment.

The CMWs shared hostel accommodation with other trainees (nursing and allied) at the school and most of them were abusive and abrasive with them.

*“The LHV students used to verbally abuse us. We were made to clean the hostel, wash their bathrooms, … They used to say that if they (MNCH program) are giving you free training then why don’t they arrange for a separate hostel for you all”* (CMW)

The CMWs deployed in field faced similar problems and were abused by both community members and local TBAs. The local community mistreated the CMWs and verbally abused them when they visited households of pregnant women.

*“Once I went to one pregnant lady’s house, she insulted me and said ‘why do you come daily? We could have put a board and started working as CMW? Who gave you permission? If you can conduct the deliveries then you should be given a job in the hospital. Don’t come to my house again or I will report you’. Sometimes they say things that we feel so bad about and often think of not going to their homes again.”* (CMW)

Another important issue raised by all community health workers was of harassment by men. Our findings indicate that this was a widespread problem and had serious implications on mobility and motivation of community based health workers. Harassment was mainly perpetrated by male members of community, usually while they were visiting community households. Respondents reported that while moving around in community, men make rude comments about their work and responsibilities. The midwives had shared their mobile phone numbers with the community members for use in case of emergency but this backfired and they started getting calls from irrelevant men at odd hours.

*“The LHWs asked us to give our contact numbers to the patients when they visit with us but after that they tell them not to contact us for delivery. There is another issue as well; we get wrong calls and are harassed when we give our contact number (loag hamay tang karna shuro kar daitay hain).”* (CMW)

### Competition for service provision

A fact that came up again and again during the course of our discussions with community midwives was that of competition from both trained and untrained community based maternal health care providers. The CMWs had three major competitors – public sector rural health center which gives free services, local LHV who has her own clinic setup locally and local TBAs.

The LHV is appointed at local rural health center (RHC) where she works in the mornings and has an evening private setup in her local village. The LHV has long term alliances with local LHWs who refer pregnant women to her clinic for antenatal and delivery services in return for a commission.

*“The LHS, LHW and LHV are cousins. When I visit a home, the LHV turns that family against me and tells them that I have no experience, and that she is more qualified given her years of experience. And also that she would deliver the baby for free at the RHC (Rural Health Centre)”* (CMW)

In discussions with the LHWs, it appeared that they were reluctant to refer pregnant women to the newly deployed CMWs. One reason cited was that LHWs had to refer a minimum number of deliveries to their local RHC every month. Another reason was the deficient supplies with CMWs for providing comprehensive services; LHWs were not willing to compromise their reputation. It also became apparent during discussion that some of the LHWs considered CMWs as their competitors and replacements.

With regards to the CMWs’ working relation with LHWs, they raised the issue of commissions – LHWs in some areas had demanded a payment from the CMWs in exchange for clients they refer to them.

*“Everyone has her own experience. The LHWs cooperate with those who they know. In my area I have 6 LHWs. They say that they would cooperate and refer the cases only if I give them 50% commission and said that “we refer the cases to those who give us a commission”. I tell them that people pay me very little; if I give you PKR 250 out of PKR 500 then how will I survive. Therefore they have never referred any antenatal or delivery cases to me. Whatever I have done, it was by my own efforts.”* (CMW)

In discussions with LHS, as supervisor of both the LHW and CMW, they acknowledged the rift between community based health workers.

*“When you introduce a new hen to a chicken coop, initially the older hens peck the new one but after some time, they adjust to each other (Durbay main aap nai murghi latay hain to purani murghian to ussay thongain marti hain na shuroo main phir adi ho kay theek ho jati hain)”* (LHS)

The respondents expressed that local untrained TBAs, were the other strong opponents of CMWs in rural communities. Usually, the TBA is an elderly woman and long term resident of the community she works in. They have the trust of locals who respect her advice, especially for maternal healthcare and act accordingly. Penetrating this network and gaining entry into field of services provided by the TBA was a daunting task for the young CMWs.

Another important aspect in the uptake of CMWs services was that the package of services provided by the TBA included not only conducting the delivery but also caring for the newborn, cooking, cleaning, washing and massaging the woman in the postpartum period. Another notable characteristic of TBA was that she demanded a very small fee and was willing to accept payments in kind such as food rations, clothes, etc.

*“Women come to us for antenatal and we also visit them but they don’t come to our health houses for delivery instead they call the TBA; they say the TBA takes 2 kilos of sugar, a bag of flour (atta) and only 200 rupee. She also washes our clothes, dishes and does the massage (maalish) for 40 days so she is better than you”* (CMW)

Competing against this network and flexible service package was proving difficult for the young CMWs, who said that as they were educated, trained in proper care methods for pregnant women, they could not be expected to give the same service package as a TBA.

*“I delivered babies of two women, and their TBAs refused to do usual massage after delivery saying that they should get this massage from the one (CMW) who did the delivery (Jis say delivery karwai hay us say kaho who maalish karay). There was this third pregnant woman, she did not get delivered from me, and she said our dai refused to help us at all, so I will get delivered from her.”* (CMW)

Other community health workers highlighted that local TBAs also considered the CMWs as a competitor for their business and earnings.

*“Dais are not willing to cooperate with CMWs. They say nobody can willingly give their livelihood away, if they introduce CMWs to the community then they will not come to us dais (apna nawala kaun kisi ko deta hai, hum agar tumhain saath lay kar jain gain to phir humaray paas koi nahin aayay ga).”* (LHW)

Some of the CMWs had tried to develop a collaboration with local TBA whereby the arrangement agreed upon was that CMW would do the delivery, TBA would provide other household services and they would share the earnings. This, however, did not work out as TBAs were apprehensive of being phased out of the community.

*“When I started, I asked two TBAs that I would go with them and conduct the deliveries and we can share the fee. They took me for 2 or 3 deliveries and but then started avoiding me as they thought that their business and reputation would be ruined as people would refuse to pay them.”* (CMW)

### CMWs’ aspirations for facility based jobs

CMWs had joined the program to earn a living and support their families. They expected that after training they would have a job at a government health facility, with support and resources for providing maternal health care in community.

CMWs repeatedly emphasized that a government job would provide them a career path along with the requisite support for carrying out their duties such as medicines, resuscitation equipment and transport for moving around in the community. It would also enable them to have a private practice after work hours.

Another reason for desiring a facility based job, as cited by CMWs, was the community’s perception that qualified health professionals work in facilities rather than going door to door and in a facility based job CMWs would get that recognition and respect from community members as maternal health care providers. Some aspired for a status similar to that enjoyed by the LHV; others were interested in completing secondary school education to be eligible for a degree in nursing.

Community midwives also expressed a strong desire for respect from community members as trained and qualified maternal health providers, and recognition as being better than local TBA.

*“The nurses at the hospital are also young but since they are working in the hospital people respect and trust them. If we are also appointed at hospital people would have their deliveries done by us. People also don’t prefer to deliver at our clinic because we don’t have the delivery tables and necessary medicines.”* (CMW)

## Discussion

Community midwifery service is an important component of National MNCH Program and, if implemented completely, can increase access to skilled care at childbirth. This study explored the challenges and constraints faced by community based health providers in delivery of maternal health services in rural Attock. The MNCH program had initiated midwifery trainings in the year 2006 and first batch of trained midwives were deployed in 2009; this study was conducted at the time when CMWs had been working in community for two years. At the time, devolution of Federal Ministry of Health was imminent and fate of vertical programs was still undecided. Midwifery Report 2011 recognizes lack of national policy and strategy as a major block to improving maternal care with in the resource constrained health systems in developing countries [32]. At the time of the study devolution being in transition, weak planning and management capacity at district level has been highlighted in our study as the main factor hindering establishment of CMWs in communities. International literature too has identified a triple gap, similar to our findings, comprising of competencies of the CMWs, coverage and access to manage the estimated number of pregnancies, subsequent births and related complications. In most developing countries there are not enough qualified midwives and other providers with midwifery competencies to manage the estimated antenatal, perinatal and postnatal care. Midwifery report of 2011 has also highlighted poor focus of educational institutions, regulatory bodies and midwifery association on quality of care [32]. Our study brings out this gap and is reflected in poor credibility of CMWs; they have no equipment and supplies to offer services and there is absence of referral mechanisms for cases they refer to government run facilities.

### CMWs - not adequately prepared

The training of the CMWs has some systemic and structural flaws, the result of which is a community health worker who has technical knowledge but skills for working in community setting are underdeveloped. At the start of this initiative, the program failed to give CMWs comprehensive information about the scope of work. Poorly resourced training institutions lacked appropriately trained tutors, non-functional clinical training facilities and almost no exposure to community settings. This study highlighted these gaps in training of CMWs and the result was a sub optimally trained health worker, not prepared to work in the resource poor, community setting. The facilities at training school and hostel did not have provisions for housing and accommodating children of married women, with the result that most of married women left the program. Young unmarried girls were selected and age and marital status of the community midwives proved to be a hurdle for young health workers. Community members were reluctant to trust them for providing maternal health care services as they perceived them to be naïve and irresponsible. This is similar to findings of other studies conducted in Pakistan underscoring the deficiencies in knowledge and skill levels of the community midwives [33]. Quantitative assessments of community midwives and their training schools from several districts of Pakistan revealed that the CMWs began their work with inadequate knowledge and skills. A skills assessment of 106 CMWs from six rural districts showed that trained CMWs lack knowledge of and basic skills relevant to maternal and newborn care [34]. Similarly, an evaluation of 22 training institutes and 25 allied hospitals from across Pakistan showed that the CMW program has adequate infrastructure but has issues in recruiting appropriate candidates, tutors are insufficient and lack required competencies for training CMWs and the program lacks an effective administrative structure for managing trained CMWs [35].

### Competition in field for maternal care services

The CMWs’ deployment was not carefully planned with the result that community midwives were unable to establish themselves in the community. Maternal care services were being provided by multiple providers which included local public sector facility (RHC), private providers such as a lady doctor and LHV and traditional birth attendant (Dai). The study highlighted that, so far, CMW has been unable to develop a niche for herself and has also been unable to develop a working relationship with any of them. One reason could be that that the CMW has not been linked with the public sector facility, as this would have given her requisite field support such as referral site for complicated cases, access to medicines and equipment. Another reason may be that CMWs are not skilled or trained in initiating a partnership with other maternal care providers. The other significant field competitor is the Dai, who has a long standing link with local community, and is providing household services to the new mother in post natal period and takes payments in-kind as well.

The program relied very much on cooperation of LHWs for success of CMWs in community and for referrals of pregnant women. However, study results show that LHWs were not facilitating them adequately. One reason for this could be lack of linkages between MNCH and LHW program. The LHWs and LHSs highlighted that they had not been given a written directive from their managers to assist the CMWs in field. Also, LHWs perceived the CMWs as a potential threat to their jobs and saw them as replacing them as their future replacements. Other studies conducted in Pakistan (Punjab, Sind and Azad Jammu Kashmir) also report antagonism towards CMWs by dais, preference for private providers and availability of free services in civil sector hospitals as major barriers for CMWs to establish themselves as skilled, community based maternal care providers [21,22,36]. As for referring pregnant women to CMWs for services, there were conflicting interests in built within the health system; LHWs’ performance was assessed on number of pregnant women they referred to the local public health facility for delivery and did not include referrals made to CMWs.

### Weak district health system

Results of the study also show that the district health system has a limited administrative and financial capacity to effectively implement program policies and monitor CMWs. The MNCH program is a vertical program and has a top-down management approach; district managers are housed in district health system but are governed by central management. Roles of district officials are not clearly defined; EDO (Executive District Health Officer) has all administrative and financial authority at district level but finances for the program are managed by provincial and federal authorities. This is evident from the fragmented field support and supervision provided to CMWs including delayed transfer of stipends. MNCH program all over Pakistan, as reported by other studies, is being affected by similar fragmentation and management issues, especially lack of trained tutors, supervisors and inappropriate training sites [22,35]. This could be a result of lack of ownership of the program and CMWs by key stakeholders especially District and Provincial Health systems and other public sector health infrastructure employees. Insufficient and inconsistent resource allocation is another factor contributing to the disparity as only 0.57 percent of GDP is spent on health in Pakistan [37]. Financial constraints are a major risk to the program's sustainability as evident from lack of equipment, supplies and delays in payments to deployed CMWs. Similar constraints have been reported by CMW program assessments in other regions of Pakistan [22,38].

### Hostile working environment

Working environment of CMWs was defined by the prevailing health system and local context; eventual impact of these systemic and contextual factors was on CMWs, who were finding it extremely difficult to establish themselves in community as qualified maternal health care providers and hence were demotivated to continue working in the public sector. Cultural values and context (religious and patriarchal) have a great influence on interactions and mobility of CMWs in their catchment population. A companion for field visits added to transportation costs which added to financial burden of CMWs’ households. Family members of CMWs were reluctant to support them in their work beyond the necessary service period. CMWs were thus compelled to continue to work because of the mandatory service bond. There is a need for MNCH program to develop formal linkages with other community based health care programs such as NGOs, charitable organizations, etc., for mobilizing existing resources and facilitating CMWs in delivering their responsibilities [34].

The CMWs were also faced with unwelcome, often hostile attitudes of community towards their services. This unfriendly stance of the community women and harassment by men contributed to CMWs’ lack of motivation and willingness to continue working in an unregulated, field environment. If the health system does not address their problems, there is a risk that deployed CMWs would eventually be unable to sustain themselves in community settings. The district and provincial health systems need to develop comprehensive guidelines for CMWs, provide stronger field support, develop flexible fee structure, and take more ownership of CMWs and the MNCH program in general.

This study shows that CMWs deployed in District Attock of Pakistan are inadequately trained and supported for provision of community based services; they are faced with programmatic and cultural constraints and have misperceptions about their job descriptions and support from the MNCH program.

### Limitations of the study

The culture and practices in the study district are reflective of northern districts of the Punjab only. Our findings are only generalizable to districts with similar situation and not to all rural districts of the country. By design a qualitative study does not produce generalizable results beyond the study participants. Our study, by including a diverse group of participants, has brought out a breadth of key issues affecting for efficient performance of CMWs. The aim of the study was to get insight of the barriers confronted by the CMWs in delivering their services. On the basis of our results further quantitative studies can be designed to evaluate the performance of CMW program. The teething problems highlighted by this study may be attributed to the program being in its infancy stage while preparing for imminent structural changes in management and organization at macro level associated with devolution.

## Conclusions

Community based midwives can play a vital role in reducing the burden of maternal morbidity and in achieving significant gains in improving maternal and child health, However, considering the results above, these improvements cannot be made in Pakistan unless MNCH program and public sector health system take steps to improve the prevalent situation. There is a need to revise the training curriculum of CMWs so that they are trained for provision of services in resource poor settings using equipment available in such areas. The MNCH program should also take steps to increase awareness of the rural communities about the CMWs and the services offered by them. Advocating the CMWs as trained care providers can increase the potential acceptability of the CMWs by rural communities. There is also a need to strengthen the communication skills of the CMWs, enabling them to interact effectively with the community members. The MNCH program also needs to develop and define innovative mechanisms for retaining CMWs for continued service delivery at community level. This may be achieved by developing and defining a career path for CMWs.

Community midwives are a key to achieving success in reducing maternal mortality. A well-trained, appropriately located, highly motivated and properly supervised CMW can deliver the desired results. Without addressing the issues and constraints faced by CMWs, the target of achieving universal coverage of skilled birth attendants will not be possible.

## Endnotes

^a^In Pakistan, *Tehsil* is the second-lowest tier of local government; each *tehsil* is part of larger District.

^b^Community base maternal health care providers included in the study comprised of Lady Health Workers and Supervisors, Lady Health Visitors and Community Midwives.

^*c*^*Mohalla* is the local term for households in a neighborhood.

^d^The biraadari is a social unit, the central elements of which are blood relationships, affective ties, trust and reciprocity.

## Competing interests

The authors declare that they have no competing interests.

## Authors’ contributions

MS conceived the study, coordinated and drafted the manuscript. MS and SH participated in the design of the study, performed the analysis and helped to draft the manuscript. All authors have read and approved the final manuscript.

## Pre-publication history

The pre-publication history for this paper can be accessed here:

http://www.biomedcentral.com/1471-2393/14/59/prepub
